# Environmental and Metabolic Risk Factors Linked to Gallbladder Dysplasia

**DOI:** 10.3390/metabo14050273

**Published:** 2024-05-08

**Authors:** Andrei Bojan, Catalin Pricop, Manuela Ciocoiu, Maria Cristina Vladeanu, Iris Bararu Bojan, Oana Viola Badulescu, Minerva Codruta Badescu, Carmen Elena Plesoianu, Dan Iliescu Halitchi, Liliana Georgeta Foia

**Affiliations:** 1 Department of Surgical Sciences, University of Medicine and Pharmacy Grigore T. Popa, 700115 Iasi, Romaniacatalin.pricop@umfiasi.ro (C.P.); 2 Department of Pathophysiology, University of Medicine and Pharmacy Grigore T. Popa, 700115 Iasi, Romania; maria.apavaloaie@umfiasi.ro (M.C.V.);; 3 Department of Internal Medicine, University of Medicine and Pharmacy Grigore T. Popa, 700115 Iasi, Romania; minerva.badescu@umfiasi.ro (M.C.B.);; 4 Department of Biochemistry, University of Medicine and Pharmacy Grigore T. Popa, 700115 Iasi, Romania; georgeta.foia@umfiasi.ro

**Keywords:** gallbladder dysplasia, mucins, gallbladder cancer

## Abstract

Gallbladder disorders encompass a spectrum from congenital anomalies to inflammatory and neoplastic conditions, frequently requiring surgical intervention. Epithelial abnormalities like adenoma and metaplasia have the potential to progress to carcinoma, emphasizing the importance of histopathological assessment for early detection of malignancy. Gallbladder cancer (GBC) may be incidentally discovered during cholecystectomy for presumed benign conditions, underscoring the need for a thorough examination. However, the lack of clarity regarding the molecular mechanisms of GBC has impeded diagnostic and therapeutic advancements. Timely detection is crucial due to GBC’s aggressive nature and poor prognosis. Chronic inflammation plays a central role in carcinogenesis, causing DNA damage and oncogenic alterations due to persistent insults. Inflammatory cytokines and microRNAs are among the various mediators contributing to this process. Gallbladder calcifications, particularly stippled ones, may signal malignancy and warrant preemptive removal. Molecular pathways involving mutations in oncogenes and tumor suppressor genes drive GBC pathogenesis, with proposed sequences such as gallstone-induced inflammation leading to carcinoma formation. Understanding these mechanisms, alongside evaluating mucin characteristics and gene mutations, can deepen comprehension of GBC’s pathophysiology. This, in turn, facilitates the identification of high-risk individuals and the development of improved treatment strategies, ultimately enhancing patient outcomes. Thus, in this review, our aim has been to underscore the primary mechanisms underlying the development of gallbladder dysplasia and neoplasia.

## 1. Introduction

Conditions involving the gallbladder are frequently encountered in medical practice, often requiring surgical intervention. These conditions span a wide spectrum, from congenital anomalies to inflammatory and both non-invasive and invasive neoplastic diseases. Epithelial abnormalities such as adenoma, atypia, and metaplasia can progress to carcinoma.

The range of histopathological findings in the gallbladder is broad. Observing changes like metaplasia, hyperplasia, and dysplasia can aid in detecting potential malignancy. Incidentally, gallbladder cancer (GBC) has been detected in 0.2 to 3% of all cholecystectomies performed for presumed benign conditions [1,2,3]. Hence, thorough histopathological examination of different gallbladder lesions is essential to assess their potential for malignant transformation.

The limited progress in diagnosing and managing gallbladder neoplasia over the past three decades could be attributed to a lack of comprehension of the molecular mechanisms underlying GBC’s pathogenesis [4,5].

Only a small portion of gallbladder lesions are found to be neoplastic. However, untreated chronic cholecystitis may be induced through a series of premalignant changes, a form of invasive cancer. Recent studies emphasize the importance of precursor lesions and the expression of apomucin in cholecystectomy specimens.

The presence of nonspecific symptoms often leads to delayed diagnosis of gallbladder cancer, contributing to its progression and unfavorable prognosis. Surgical treatment remains the sole potential curative therapy. Some individuals have the chance to benefit from an incidental discovery of gallbladder cancer during cholecystectomy performed for gallstones. Early detection is crucial, as late diagnosis often indicates advanced disease, lymph node dissemination, and potential recurrence post-resection. The average survival time is merely 6 months, with a 5-year survival rate of only 5%. The influenced prognosis is partly attributed to the absence of a serosal layer in the gallbladder in the proximity of the liver, facilitating hepatic invasion and metastasis. More detailed imaging techniques aid in early diagnosis [6,7,8].

Examining the environmental and metabolic anomalies of the cholecyst, immunohistochemical characteristics of mucins and the occurrence of various gene anomalies in karyokinetic abnormalities of the gallbladder and proximal mucosa could enhance comprehension of the disease’s pathophysiology and the underlying mechanisms of tumor formation. This could facilitate the identification of individuals with heightened susceptibility to neoplasia, thereby enhancing treatment strategies for better patient outcomes.

## 2. Materials and Methods

A systematic search of all published studies related to gallbladder dysplasia and neoplasia was performed in PubMed to identify the status of research on the different mechanisms of inducing lesions involved in gallbladder tissue.

Studies were excluded if they were not written in English or included single case reports.

Screening of search results was divided into three steps: review of titles and abstracts, assessment of full manuscripts for eligibility, and inclusion of studies for systematic review.

We identified 409 results from Pubmed. Studies not written in English or comprising single case reports were excluded, resulting in 62 original articles, systematic reviews and meta-analyses. Among these, one was excluded for focusing on children—see Table 1.

## 3. Detection Methods and Biomarkers in Gallbladder Neoplasia

Methods for detecting gallbladder karyokinesis typically involve employing a blend of imaging technologies and laboratory assessments. Initially, ultrasound serves as a primary imaging tool to identify gallbladder anomalies like masses or thickened walls. Further insight into the tumor’s scope and its interaction with neighboring structures is often gained through computed tomography (CT) scans and magnetic resonance imaging (MRI). Endoscopic ultrasound (EUS) offers high-resolution images of the gallbladder and surrounding tissues, aiding in the detection of smaller lesions. Additionally, certain blood markers, such as CA 19-9 and carcinoembryonic antigen (CEA), may show elevated levels in gallbladder cancer patients, though these markers lack specificity. For a definitive diagnosis, confirmation through biopsy procedures like endoscopic retrograde cholangiopancreatography (ERCP) or fine-needle aspiration (FNA) may be required. Overall, an integrated approach combining various imaging modalities, laboratory tests, and tissue sampling techniques is essential for precise detection and diagnosis of gallbladder neoplasia [6,7].

Biomarkers are pivotal in gallbladder cancer diagnosis, prognosis, and treatment. They serve as indicators aiding in disease detection and monitoring. Among these biomarkers, CA 19-9 has been extensively studied. Elevated CA 19-9 levels in blood serum are associated with advanced disease stages and poorer prognosis. Carcinoembryonic antigen (CEA) is another biomarker often elevated in gallbladder cancer patients, though its specificity is limited. Additionally, mucin glycoproteins like MUC1, MUC4, and MUC5AC, overexpressed in gallbladder cancer, are potential biomarkers indicating disease progression. Genetic and molecular biomarkers, including TP53 and KRAS gene mutations and microRNA expression alterations, show promise in identifying high-risk patients and predicting treatment response. While biomarkers offer the potential for enhancing gallbladder cancer management, further research is necessary to validate their clinical usefulness and optimize their application in clinical settings [7,8].

Novel biomarkers are increasingly recognized for their potential to transform diagnostics, prognostics, and therapeutic strategies. MicroRNAs (miRNAs), long non-coding RNAs (lncRNAs), and circulating tumor cells (CTCs) are among the innovative biomarkers being actively investigated. miRNAs, with their regulatory roles in gene expression, and lncRNAs, which are implicated in tumor initiation and progression, offer promise as diagnostic and prognostic markers. Additionally, the detection and analysis of CTCs in the bloodstream present a non-invasive avenue for monitoring disease progression and treatment response. Despite their potential, further validation and standardization efforts are crucial to establish the clinical utility of these novel biomarkers and facilitate their integration into routine clinical practice, ultimately advancing the management of gallbladder dysplasias and neoplasias and improving patient outcomes [8].

### 3.1. Risk Factors

Identifying risk factors is crucial, as it offers an understanding of the pathogenetic mechanisms underlying geographic and ethnic differences and provides valuable insights for devising prevention and treatment strategies.

### 3.2. Environmental Risk Factor

Numerous environmental exposures have proved to play a role in the development of gallbladder karyokinesis. There have been suggestions that heavy metals like nickel and cadmium might play a role, although a conclusive link has not been confirmed. Miners, who often encounter radon, have been associated with both lung and gallbladder cancer. Tobacco consumption is a recognized risk factor. Additionally, certain medications, such as methyldopa and isoniazid, have been suggested as potential contributors. The connection between oral contraceptives and gallbladder neoplasia remains uncertain [9,10].

### 3.3. Geographic Distribution

The distribution of gallbladder cancer exhibits significant geographic diversity, unlike other tumors affecting the extrahepatic biliary tract and the ampulla of Vater. Incidence rates are notably elevated in Latin America and Asia, moderately elevated in specific countries in eastern and central Europe, yet relatively low in the United States and most Western and Mediterranean European countries. Gallbladder cancer disproportionately impacts indigenous populations, as illustrated by comprehensive data from a global cancer registry covering five continents, encompassing 704.4 million individuals or 11% of the world’s population. Asia stands out as a region with heightened risk, as evidenced by elevated rates of gallbladder cancer among northern Indian females, Pakistani females, and Korean males. Ethnic disparities persist across various geographic areas [2,4,11].

### 3.4. Age

The incidence of gallbladder neoplasia tends to rise with increasing age. According to a report from Memorial Sloan–Kettering, the median age among 435 gallbladder cancer patients was 67 years. Data from the US in 2010 indicates that age-adjusted incidence rates (per 100,000) increased from 0.16/100,000 (for individuals aged 20–49) to 8.69/100,000 for those over 75 years old. The highest mortality rate documented was 5.05 per 100,000 individuals aged 75 and above [12,13].

## 4. Gallstones

Gallstones are known to be the primary risk factor for gallbladder cancer (GBC), documented in 70–98% of GBC cases in the literature. However, autopsy studies reveal that only 1–4% of individuals with gallstones develop GBC, contrasting with the occurrence rate of 0.2% in cases of acalculous cholecystitis. Gallstone characteristics significantly impact the development of gallbladder cancer. Larger stone size increases the risk; stones exceeding 3 cm are associated with a tenfold higher risk compared to smaller stones. Moreover, the type of stone may be relevant. Ethnic groups with an increased incidence of gallbladder carcinoma often show a disproportionately high prevalence of cholesterol gallstone disease. This link is likely due to gallstones causing irritation of the local mucosa and persistent inflammation, potentially exacerbated by the production of carcinogens such as secondary bile acids within the region [14].

## 5. Chronic Inflammation

Chronic inflammation is intricately associated with the process of malignant transformation, representing a significant factor in carcinogenesis. Persistent or recurrent inflammatory insults have deleterious effects, causing damage to deoxyribonucleic acid (DNA), triggering repeated attempts at tissue repair and proliferation, releasing cytokines and growth factors, and thereby predisposing cells to karyokinetic changes. Several factors, including nuclear factor kappa B, reactive oxygen and nitrogen species, inflammatory cytokines, prostaglandins, and certain micro-ribonucleic acids (micro-RNAs), could potentially exhibit oncogenic properties. These factors may impact various cellular processes such as cell proliferation, apoptosis (cell death), DNA mutation rates, DNA methylation, and angiogenesis. Consequently, the presence of cholelithiasis and the associated repetitive trauma leading to chronic cholecystitis may represent the mechanism through which neoplasia develops gradually over the course of many years [9,15].

Persistent inflammation may cause the deposition of calcium in the wall of the gallbladder as well. In a recent systematic review comprising 340 patients with gallbladder calcifications, findings indicated that 21% of them were diagnosed with gallbladder malignancy. Notably, only those presenting with stippled calcifications are considered precancerous, while cases with transmural calcifications are less likely to be associated with carcinoma. As a result, gallbladders exhibiting partial calcification should be preemptively removed [15,16].

### Chronic Inflammation Due to Pancreaticobiliary Maljunction

Pancreaticobiliary maljunction (PBM) is a congenital anomaly where the pancreatic and bile ducts anatomically join outside the duodenal wall. Diagnosis of PBM requires demonstrating an abnormally long common channel or an unusual connection between the pancreatic and bile ducts, typically through direct cholangiography methods (such as endoscopic retrograde cholangiopancreatography, transhepatic cholangiography, or intraoperative cholangiography) or magnetic resonance cholangiopancreatography (MRCP). The carcinogenic mechanism in PBM seems to be associated with the continuous reflux of pancreatic juice into the biliary tract. This reflux, possibly due to increased intraductal pressure or bacterial infection, triggers the activation of proteolytic pancreatic enzymes and phospholipase A2 within the biliary tract. Phospholipase A2, known for its potent destructive action, converts bile lecithin into lysolecithin, causing significant damage to cell membranes. PBM patients exhibit elevated levels of secondary bile acids, particularly taurodeoxycholic acid, and mutagenic substances that cause DNA damage, observed both clinically and in experimental animal models. Exposure to such harmful substances accelerates the cell cycle, resulting in adverse effects on the epithelium and DNA damage [17].

## 6. Infectious Markers

A clear risk factor for malignancies of the biliary tract is induced by chronic bacterial cholangitis. *Salmonella* species (*S. typhi* and *S. paratyphi*) and Helicobacter species are noteworthy. Approximately 6% of individuals who are carriers of typhoid develop gallbladder neoplasia, marking a twelvefold increase in the subsequent risk. Bacterial colonization may facilitate a karyokinetic transformation by altering bile constituents through processes such as bacterial hydrolysis of primary biliary acids to form carcinogens and/or the action of β-glucuronides. Furthermore, the process of malignant transformation might be impacted by chronic inflammation itself and/or alterations in tumor suppressor genes (such as tumor protein 53 [p53]) or proto-oncogenes (for instance, mutations of Kirsten ras oncogene homolog (K-ras)) [17,18,19].

## 7. Metabolic Risk Factors

### 7.1. Obesity and Insulin Resistance

From a clinical standpoint, obesity stands as a firmly established significant risk factor for the development of gallstones. While the precise biological pathways through which obesity might contribute to carcinogenesis are not fully elucidated, several plausible mechanisms have been proposed. These factors encompass insulin resistance leading to chronic hyperinsulinemia, elevated production of insulin-like growth factors (IGF), and increased levels of steroid hormones. Insulin plays a pivotal role in metabolic syndrome. The karyokinetic effect of insulin could be attributed to direct interactions with insulin receptors (IR) on (pre)neoplastic target cells, or it may result from changes in endogenous hormone metabolism due to hyperinsulinemia. Insulin promotes the production and activity of IGF-I and affects the synthesis and availability of sex hormones, including androgens, progesterone, and estrogens [20,21].

Insulin and IGF-I transmit signals by binding to their respective receptors, insulin receptors (IRs), and IGF-IR. This binding fosters cellular proliferation and suppresses apoptosis in various tissue types, potentially establishing a milieu conducive to tumor formation. The main cellular survival pathway triggered within the IGF-I axis is the phosphatidylinositol 3 kinase/Akt (PI3K/Akt) signaling pathway. This pathway regulates essential cellular functions such as growth, proliferation, and glucose metabolism. This activation leads to the phosphorylation of numerous downstream targets, which include the proapoptotic Bad protein and nuclear factor (NF) κB. Consequently, this impedes apoptosis and enhances cellular survival signaling pathways.

The PI3K/Akt pathway also stimulates protein synthesis and cellular growth by activating mammalian target of rapamycin (mTOR). mTOR, a conserved Ser/Thr kinase, regulates cell growth and metabolism and has recently been implicated in the carcinogenesis of several cancers. Additionally, IGF-I triggers the activation of the Ras/Raf/mitogen–activated-protein-kinase (MAPK) pathway, primarily promoting cellular proliferation through downstream target proteins. Recent studies suggest that various factors altered in obesity, such as increased levels of blood insulin, leptin, TNFα, IL-6, and decreased adiponectin, may all enhance the activity of the PI3K/Akt signaling pathway, thereby contributing to carcinogenesis. Adiponectin, an adipokine primarily released by visceral fat adipocytes, demonstrates an inverse relationship with BMI. It functions as an insulin-sensitizer and exhibits significant anti-inflammatory and antiangiogenic effects. Research findings indicate that decreased adiponectin levels, termed hypoadiponectinemia, are linked to cholesterol gallstones in humans. This implies that hypoadiponectinemia may contribute to the development of gallstone disease. Even more, mounting evidence suggests that obesity is inevitably linked to the accumulation of fat in the gallbladder, leading to a condition known as lipotoxic cholecystopathy or fatty gallbladder disease. The heightened deposition of fat in the gallbladder can exacerbate chronic inflammation locally, resulting in an abnormal wall structure and reduced contractility [22,23].

### 7.2. Genetic Risk Factors

The molecular progression of pathogenesis arises from an accumulation of mutations, ultimately leading to the onset of malignancy. Common genetic alterations affect oncogenes, tumor suppressor genes, microsatellite stability, and gene promoter methylation.

The pathways that may lead to karyokinesis include:

1. the inflammatory status induced by gallstones, followed by p53 mutation and eventual carcinoma;
2. K-ras point mutations play a role in the progression from atypical epithelium to carcinoma, as seen in the hyperplasia–carcinoma sequence in patients with an anomalous junction of the pancreaticobiliary duct;
3. the potential emergence of neoplastic foci in gallbladder polyps due to K-ras mutations;
4. tumor growth is further fuelled by neovascularization, facilitated by a synergistic interaction between p53 mutation and increased vascular endothelial growth factor expression [24,25,26].

When assessing the potential risk of gallbladder karyokinesis, it is crucial to comprehend the association between gallbladder polypoid lesions and GBC, enabling their prudent and timely management. Gallbladder polyps have a population prevalence of approximately 5%, constituting 2–12% of cholecystectomy specimens. Of particular significance are low-grade biliary tract intraepithelial neoplasia (LG-BilIN) and high-grade biliary tract intraepithelial neoplasia (HG-BilIN). Morphological investigations have revealed a histological continuum from LG-BilIN to gallbladder cancer (GBC), either with or without adjacent HG-BilIN.

Low-grade biliary tract intraepithelial neoplasia (LG-BilIN) is a significant pathological entity characterized by dysplastic alterations in the epithelial lining of the biliary system. These lesions typically exhibit mild cytological abnormalities and architectural changes, including glandular distortion and minimal cellular stratification. While LG-BilIN is histologically categorized as benign, its importance lies in its potential to progress to invasive carcinoma, notably gallbladder cancer (GBC). Research indicates a gradual advancement from LG-BilIN to more severe forms of biliary intraepithelial neoplasia and ultimately to invasive cancer, underlining the necessity of identifying and monitoring these lesions for early intervention and prevention of malignant transformation.

High-grade biliary tract intraepithelial neoplasia (HG-BilIN) is characterized by significant dysplastic alterations in the epithelial lining of the biliary system. Unlike its low-grade counterpart, HG-BilIN displays prominent cytological abnormalities and architectural distortions, including marked cellular stratification and disruption of normal glandular structure. Although still categorized as a precursor lesion, HG-BilIN carries a heightened risk of progressing to invasive carcinoma, particularly gallbladder cancer (GBC). Studies have highlighted a clear histological continuum from HG-BilIN to invasive cancer, emphasizing the importance of early detection and intervention to mitigate the potential for malignant progression. Vigilant monitoring and timely management of HG-BilIN lesions are crucial for preventing the development of advanced biliary tract malignancies [27].

A recent study has shown that GBC, LG-BilIN, and HG-BilIN exhibited comparable mutation rates. However, tumor samples from younger patients displayed significantly fewer mutations compared to those from older patients, irrespective of tumor type. Microsatellite instability (MSI) status was further quantified in these tumor samples based on a large set of selective microsatellite loci24, revealing no significant difference among tumor types [27].

In total, 16 potential mutation drivers were identified. Among them, CTNNB1 and ARID2 mutations were found in 45% of patients, whereas TP53 and ERBB3 mutations were present in 25% of patients. Interestingly, GBC samples did not exhibit a higher frequency of driver mutations compared to LG- or HG-BilIN. In terms of specific mutations, four different CTNNB1 mutations were detected. These included p.T41I, p.S45F, p.S33C, p.S45F, and p.K335T in LG-BilIN and GBC. All these CTNNB1 mutations were likely deleterious. Notably, CTNNB1 mutations have been linked to altered β-catenin activity associated with liver tumor progression, underscoring the pivotal role of β-catenin activity driven by CTNNB1 mutations in cellular transformation during GBC tumorigenesis [27,28,29].

ARID2 mutations were detected in 45% of patients, with p.Q916 being consistently present, indicating a loss-of-function ARID2 as a driving factor in these individuals. In the remaining 35% of patients, ARID2 mutations were exclusively identified in samples of gallbladder cancer (GBC) or biliary intraepithelial neoplasia (BilIN). Among patients with ERBB3 mutations, one mutation was shared across all three samples, while others had mutations only in BilIN samples. SMARCA4 mutations were observed solely in GBC samples, suggesting acquisition at a later stage of GBC development. Notably, significant copy-number gain of ERBB2 and significant copy-number loss of CDNK2A were noted in GBC samples, potentially contributing to GBC tumorigenesis. These findings collectively shed light on potential driver somatic alterations involved in the development of adenoma/dysplasia-related GBC [27,28,29,30].

Loss of heterozygosity (LOH) is a frequent event in cancer, and its accumulation has been linked to gallbladder carcinogenesis. Interestingly, J. Lin et al. observed that gallbladder cancer (GBC) samples in the BilIN-independent category exhibited a higher frequency of LOH compared to those in the BilIN-dependent category. Based on these discoveries, we have formulated an evolutionary model outlining the development of gallbladder cancer (GBC), where the extent of loss of heterozygosity (LOH) events and somatic mutations accrued during the early phases of gallbladder tumor initiation plays a pivotal role in shaping the subsequent trajectory of carcinoma evolution. In the BilIN-independent category, extensive loss of heterozygosity (LOH) and mutation events occur initially, creating a “cancerous” setting within the shared ancestor of LG-BilIN/HG-BilIN/GBC. With the establishment of cancerous potential in this environment, GBC tends to diverge earlier and evolve more autonomously from LG-BilIN and HG-BilIN, resulting in a higher number of clones during the microevolutionary process. Conversely, in the BilIN-dependent category, there is a reduced frequency of LOH events and somatic mutations initially, resulting in a “neoplastic” environment within the common ancestor [31,32,33].

KRAS plays a pivotal role in numerous signal transduction mechanisms and associated pathways. Gallbladder dysplastic tissue has been reported to exhibit various pathogenic mutations in the KRAS oncogene. Notably, mutations in the KRAS gene predominantly affect codons 12, 13, and 61 in GBC. Some studies have shown that mutations in KRAS codon 13 are more prevalent (approximately one-third) than those in codons 12 and 61. However, several other studies have failed to detect any mutations in this gene. Previous studies have linked an anomaly known as an anomalous arrangement of the pancreatico-biliary duct with the presence of gallbladder cancer, as patients with this anomaly exhibit a higher frequency of KRAS gene mutations compared to those without the anomaly [34,35].

TP53 is a widely acknowledged tumor suppressor gene with diverse mechanisms of anticancer action, pivotal in upholding genome integrity, fostering apoptosis, ensuring genomic stability, and restraining angiogenesis, among other functions. Dysfunction of TP53 permits the uncontrolled survival of genetically impaired abnormal cells, paving the way for potential neoplastic conversion later on. In gallbladder cancer, the majority of TP53 mutations manifest as missense mutations, resulting in the production of a dysfunctional protein with an extended lifespan. Reports from the literature indicate TP53 gene mutations in approximately 27% to 70% of gallbladder carcinomas. Pathogenic mutations affecting various codons of the TP53 gene have been documented. Molecular studies have elucidated that mutations in exons 5 and 8 of the TP53 gene disrupt its regulatory functions [35,36].

The oncogene c-erb-B2, a homolog of the epidermal growth factor receptor, encodes a protein with tyrosine kinase activity. Immunohistochemical analysis has indicated positive expression of c-erb-B2 in 10% to 46% of gallbladder cases. However, previous reports have shown its absence in dysplasia or adenomas. Studies utilizing animal models, particularly transgenic mice, have illustrated that overexpression of erbB2 in the basal layer of the biliary tract epithelium leads to the development of gallbladder cancer in all mice (100%). Additionally, positive HER2/neu expression was identified in 28% of gallbladder cancers, demonstrating a direct correlation with advanced cancer stages. This suggests a relationship between oncogene expression and gallbladder cancer progression. In an Indian study, c-erb-B2 was frequently expressed in well-differentiated and stage II to IV gallbladder cancer cases, accounting for approximately 9.4% of cases. A recent report revealed HER2/neu overexpression in 14% of advanced gallbladder cancer cases, indicating potential benefits from HER2/neu pathway inhibitors. Therapeutic targeting of the EGFR/HER2 pathways has been shown to enhance the anti-proliferative effects of gemcitabine in the biliary tract and gallbladder carcinomas. These findings imply that c-erb-B2 expression could serve as an indicator of poor prognosis [37,38,39].

Understanding the DNA methylation patterns of gallbladder tumors holds significant promise as biomarkers to refine diagnosis and prognostic information, thereby aiding in appropriate therapeutic decision-making. Hypermethylation in gene promoter regions is a prevalent epigenetic mechanism leading to the inactivation of tumor suppressor genes.

A notable research study has established a significant association between methylation patterns and patient survival in gallbladder cancer. This investigation, involving a series of 109 cases of advanced gallbladder cancer, revealed that methylation of genes like p73, MGMT, and DCL1 was notably linked to patient survival. However, genes such as CDH13 and FHIT did not exhibit a significant correlation with patient survival in gallbladder cancer. Through multivariate analysis, the MGMT gene emerged as an independent prognostic factor for survival, highlighting the pivotal role of epigenetic processes in gallbladder carcinogenesis. Recent reports have emphasized the importance of promoter methylation in specific genes, including CDH1, CDKN2A-p16, REPRIMO (a tumor suppressor gene family), and UCHL1 (also known as PGP9.5) in the development of gallbladder cancer [40,41,42].

A study conducted by Wang et al. from China proposed the concept of CCK-induced impaired gallbladder emptying in individuals with gallstones. To date, most identified candidate genes are linked to classical rate-limiting enzymes and proteins involved in lipid metabolism, steroidogenesis, lipid transport, bile acid synthesis, bile canalicular transport, gallbladder contractility, cell cycle regulation, DNA repair mechanisms, and inflammatory pathways. Nevertheless, independently replicated studies in gallbladder cancer (GBC) remain scarce, with only a few, such as OGG1rs1052133, TP53rs1042522, GSTM1 null polymorphism, and CYP1A1rs1048943 polymorphism, having been investigated. Given the limited number of studies, definitive conclusions cannot be reached, emphasizing the urgent requirement for additional exploration of genes linked to GBC susceptibility [43,44]—see Table 2.

## 8. The Role of Mucins in Gallbladder Lesions

The production of mucin by liver tumor cells has traditionally been linked to biliary differentiation. In the initial classification, mucin-producing tumors were divided into three types: papillary cholangiocarcinomas (CCs), biliary cystadenomas, and biliary cystadenocarcinomas. Pioneering comparative studies between mucin-producing CCs and non-mucin-producing CCs revealed that the former occur in 13% of cases and are associated with longer survival times. It has been confirmed that hepatobiliary mucinous cystadenoma and cystadenocarcinomas act as precursors to biliary tract tumors that excessively produce and/or secrete mucins. Consequently, cystadenomas and cystadenocarcinomas have been reclassified as “cystic intraductal papillary neoplasm of the intrahepatic bile duct” (M-IPNB, IPMN), “biliary intraductal tubulopapillary neoplasms” (b-ITPN), and “hepatic (biliary) mucinous cystic neoplasms” (MCNBs, MCN-L). The presence of an ovarian-like stroma (OLS) has been identified as a defining feature for the diagnosis of MCN-L.

The classical intraductal papillary neoplasm of the bile duct (IPNB) shares largely similar features with intraductal papillary mucinous neoplasm (IPMN). Consequently, the presence of gross mucin is common in type 1 IPNB (approximately 80%), while it is relatively rare in papillary cholangiocarcinoma (approximately 10%). It is important to highlight that the concept of IPNB remains somewhat unclear, necessitating ongoing research to further elucidate its pathological characteristics as previously described. 

Cholangiocarcinoma, presently designated as type 2 intraductal papillary neoplasm of the bile duct (IPNB), is anatomically categorized into perihilar and distal types of intrahepatic cholangiocarcinoma (iCC). Among iCCs, both hilar carcinoma (referred to as Klatskin tumor) and peripheral cholangiocarcinoma can be distinguished, with both types previously observed to produce mucin [45,46,47].

The WHO 2010 classification identifies four distinct phenotypes of intraductal papillary neoplasms of the bile ducts (IPNBs): gastric, intestinal, pancreatobiliary, and oncocytic. It recommends the analysis of specific mucin expressions (MUC1, MUC2, MUC5AC, and MUC6), along with the assessment of architectural and cell differentiation patterns, to ensure accurate classification. However, some sources simplify this classification to just two immunophenotypes of IPNBs: the “pancreatic” phenotype, which closely resembles intraductal papillary mucinous neoplasm (IPMN), and the “non-pancreatic” phenotype, which often presents with high-grade dysplasia resembling papillary cholangiocarcinoma.

In the 2018 classification, type 1 intraductal papillary neoplasms of the bile duct (IPNB) predominantly display gastric or oncocytic phenotypes, although pancreatobiliary and intestinal phenotypes are also evident. Type 2 IPNB, characterized by papillary cholangiocarcinoma, typically presents with a pancreatobiliary or intestinal phenotype. While type 1 IPNB commonly exhibits a high level of mucin production, the absence of mucin overproduction does not necessarily exclude classification into this type.

Immunophenotype profiling of various mucinous biliary tract cancers (BTCs) revealed that the gastro-intestinal phenotype (MUC2+, MUC5AC+, and MUC6+) was more prevalent in mucinous cystic neoplasms of the liver (MCN-L) and intraductal papillary mucinous neoplasms (IPMN) than in benign mucinous cystadenomas. Moreover, the expression of MUC1, MUC2, MUC5AC, and MUC6 was notably more frequent in IPMN than in MCN-L. Differential expression of MUC1 was solely observed in malignant cases of both tumor types.

Further studies have corroborated the higher mucin production observed in intraductal papillary neoplasms of the bile ducts (IPNBs) (presently categorized as type 1 IPNB) compared to papillary cholangiocarcinomas (type 2 IPNB). Notably, IPNBs (type 1) were found to exclusively harbor gastric-type and oncocytic-type tumors. Significant differences in the expression of MUC1, MUC2, and MUC6 were noted between both types of IPNBs and non-papillary cholangiocarcinomas [48,49,50].

The observed patterns of gross mucin expression correspond to one of the primary criteria used in IPNB classification, while studies focusing on specific apomucins have allowed for a more nuanced differential diagnosis of both subtypes. In another study comparing various cholangiocarcinomas, it was found that approximately 61% of intrahepatic cholangiocarcinomas (ICCs) exhibited exclusive features of mucin-producing cholangiocarcinomas (M-CCs).

Implications regarding mucin types, particularly MUC1 and MUC5AC, have emerged in the context of malignant tumor development, impacting the biological characteristics and advancement of gallbladder adenocarcinoma. MUC1 expression acts as an indicator of neoplastic transformation and extension within the gallbladder epithelium. Furthermore, MUC1 has been linked to tissue invasion, lymphatic metastasis, and the emergence of a non-papillary carcinoma phenotype. Conversely, in digestive adenomas and dysplasia, MUC5AC levels typically decline, while MUC1 levels tend to rise.

Immunohistochemistry is a commonly utilized method for assessing MUC1 and MUC5AC expression levels. Multiple studies have suggested that MUC1 expression increases as gallbladder lesion severity progresses from hyperplasia to carcinoma in situ, whereas MUC5AC expression decreases with escalating lesion severity. Specifically, MUC1 expression exhibits a significant elevation in gallbladder adenocarcinoma compared to chronic cholecystitis, while MUC5AC expression tends to diminish in neoplastic conditions relative to chronic inflammation.

One study described in the literature revealed lower MUC1 expression in T1 stage tumors compared to higher stages. They did not find a correlation between MUC1 expression and histopathologic type and grade in their study but noted an association between angio-lymphatic invasion and depolarized MUC1 expression. In our study, high MUC1 expression was correlated with less differentiated tumors. In a prior study by Xiong L et al., the correlation of MUC1 and MUC5AC in gallbladder adenocarcinoma was examined, revealing a link between MUC1 expression and T-stage (*p* < 0.01).

Moreover, heightened MUC1 expression or diminished MUC5AC expression correlated with reduced overall survival. Additionally, reduced MUC5AC expression emerged as an independent prognostic indicator. In gallbladder carcinoma, MUC1 expression may signify histological dedifferentiation, heightened proliferative activity, and invasiveness [51,52,53].

These findings underscore that while gallbladder specimens are frequently encountered in surgical pathology, cases of gallbladder cancer are relatively infrequent. It can be inferred that the expression of MUC1 and MUC5AC may be closely linked to the pathogenesis of neoplastic and non-neoplastic gallbladder lesions.

## 9. Novel Aspects Regarding the Involvement of Telocytes in Gallbladder Lesions

Understanding gallbladder motility has been associated with the discovery of telocytes (TCs), a unique cell population. Gallbladder (GB) motility encompasses the processes of bile storage, concentration, and delivery. Multiple intricate factors regulate GB motor functions, including extrinsic and intrinsic innervation, humoral factors, and neuropeptides. The coordinated contractions of the muscular layers of the GB wall lead to GB emptying. The depolarization of gallbladder smooth muscle (GBSM) relies on the activation of regular depolarization-repolarization potentials known as slow waves (SWs). These rhythmic SWs, governing GBSM contraction, involve various cell types, such as smooth muscle cells (SMCs), GB neurons, telocytes (TC), and specialized pacemaker cells referred to as interstitial cells of Cajal (ICC). These interstitial (stromal) cells—telocytes—have been identified in various tissues and organs, including the human gallbladder. TCs are characterized by their small cell body, typically measuring between 9 and 15 μm, and possess one to five extremely long and slender telopodes (Tps). Telopodes consist of alternating segments known as podomers (~80 nm) and podoms (250–300 nm) and are interconnected through homo- and heterocellular junctions, forming intricate three-dimensional networks [54]. Previous research has revealed a significant decrease in the density of c-Kit positive TCs in the gallbladder wall among patients with cholelithiasis. TCs have garnered interest for their potential involvement in cancer development and progression. While specific mechanisms are still being elucidated, emerging evidence suggests that TCs may contribute to GBC through various means. TCs have been implicated in modulating the tumor microenvironment, influencing cell-to-cell communication, and promoting angiogenesis, all of which can impact tumor growth and metastasis. Additionally, TCs have been shown to interact with immune cells and may play a role in immune responses within the tumor microenvironment. Further research is needed to fully understand the precise role of TCs in GBC and their potential implications for diagnosis, prognosis, and treatment strategies [55].

## 10. Conclusions

In this review, we offer a thorough examination of the environmental, metabolic, molecular, and genetic factors contributing to gallbladder lesions and subsequent malignancies. The molecular and cellular mechanisms are increasingly recognized as significant contributors to the intricate pathogenesis of gallbladder lesions. Future studies should aim to bridge the gap between findings observed in human tissue and animal models, allowing for the derivation of clinically relevant conclusions regarding the formation of gallbladder anomalies and the potential pharmacological intervention to block pathogenic pathways.

## Figures and Tables

**Table 1:** This table provides a clear summary of the number of studies at each step of the process, from the initial Pubmed results to the final selected studies meeting the specified criteria.

Steps	Number of Remaining Studies
Initial Pubmed results	409
Excluded: not written in English or comprising single case reports	347
Selected systematic reviews and meta-analyses plus original articles	62
Excluded: studies focusing on children	61
Final results	61

**Table 2:** Genetic pathogenesis of gallbladder lesions.

Topic	Summary	Reference
Molecular Pathogenesis of Gallbladder Cancer	- genetic alterations: oncogenes; tumor suppressor genes; microsatellite instability; gene promoter methylation.	[24,25,26]
	- proposed carcinogenic pathways: gallstone-induced inflammation; K-ras mutations; neoplastic foci in gallbladder polyps.	
Gallbladder BilIN and Gallbladder Cancer Risk	- LG-BilIN and HG-BilIN—premalignant tendencies. - histological progression from LG-BilIN to GBC.	[27]
(LOH) in Gallbladder Cancer	- higher frequency in BilIN-independent GBC samples. - shape the trajectory of carcinoma evolution.	[31,32]
KRAS Mutations in Gallbladder Cancer	- mutations in codons 12, 13, and 61—prevalent in GBC. - codon 13 mutation frequency—higher in North India. - can correlate with anomalous arrangement of the pancreatico-biliary duct.	[34]
TP53 Mutations in Gallbladder Cancer	- are common in later stages of GBC. - missense mutations result in dysfunctional protein production. - reported in 27% to 70% of GBC cases. - mutations in exons 5 and 8 disrupt TP53 regulatory functions.	[36]
c-erb-B2 (HER2/neu) Expression in Gallbladder Cancer	- is observed in 10–46% of gallbladder cases. - overexpression in transgenic mice leads to GBC development. - positive HER2/neu expression in advanced cancer stages.	[38]
DNA Methylation Patterns in Gallbladder Cancer	- DNA methylation patterns—potential biomarkers in diagnosis and prognosis. - methylation of p73, MGMT and DCL1 linked to patient survival. - promoter methylation of CDH1, CDKN2A-p16, REPRIMO, and UCHL1 implicated in GBC development.	[41,42]
Genetic Studies and GBC Susceptibility	- OGG1rs1052133, TP53rs1042522, GSTM1 null polymorphism, and CYP1A1rs1048943 polymorphism are under investigation. - further exploration is needed.	[44]

## Data Availability

The data presented in this study are available on request from the corresponding authors. The data are not publicly available due to privacy.
